# B’More healthy: retail rewards - design of a multi-level communications and pricing intervention to improve the food environment in Baltimore City

**DOI:** 10.1186/s12889-015-1616-6

**Published:** 2015-03-24

**Authors:** Nadine Budd, Alison Cuccia, Jayne K Jeffries, Divya Prasad, Kevin D Frick, Lisa Powell, Fred A Katz, Joel Gittelsohn

**Affiliations:** Center for Human Nutrition, Bloomberg School of Public Health, Johns Hopkins University, 615 N. Wolfe Street, Baltimore, MD 21205 USA; Army Institute of Public Health, Health Promotion & Wellness Portfolio, USAPHC, ATTN: MCHB-IP-H, 5158 Blackhawk Road, Aberdeen Proving Ground, Aberdeen, MD 21010 USA; The Gillings School of Global Public Health at the University of North Carolina at Chapel Hill, 135 Dauer Drive, Chapel Hill, NC 27599 USA; Joseph Stokes, Jr. Research Institute, The Children’s Hospital of Philadelphia, Philadelphia, PA USA; Carey Business School, Johns Hopkins University, 100 International Drive, Baltimore, MD 21202 USA; Division of Health Policy and Administration, School of Public Health, University of Illinois at Chicago, Chicago, IL USA

**Keywords:** Obesity, RCT, Food stores, Food access, Pricing, Intervention, Low-income, Urban

## Abstract

**Background:**

Low-income black residents of Baltimore City have disproportionately higher rates of obesity and chronic disease than other Maryland residents. Increasing the availability and affordability of healthy food are key strategies to improve the food environment and can lead to healthier diets. This paper describes B’More Healthy: Retail Rewards (BHRR), an intervention that tests the effectiveness of performance-based pricing discounts and health communications, separately and combined, on healthy food purchasing and consumption among low-income small store customers.

**Methods/design:**

BHRR is 2x2 factorial design randomized controlled trial. Fifteen regular customers recruited from each of 24 participating corner stores in Baltimore City were enrolled. Food stores were randomized to 1) pricing intervention, 2) communications intervention, 3) combined intervention, or 4) control. Pricing stores were given a 10-30% price discount on selected healthier food items, such as fresh fruits, frozen vegetables, and baked chips, at the point of purchase from two food wholesale stores during the 6-month trial. Storeowners agreed to pass on the discount to the consumer to increase demand for healthy food. Communications stores received visual and interactive materials to promote healthy items, including signage, taste tests, and refrigerators. Primary outcome measures include consumer food purchasing and associated psychosocial variables. Secondary outcome measures include consumer food consumption, store sales, and associated storeowner psychosocial factors. Process evaluation was monitored throughout the trial at wholesaler, small store, and consumer levels.

**Discussion:**

This is the first study to test the impact of performance-based pricing and communications incentives in small food stores, an innovative strategy to encourage local wholesalers and storeowners to share responsibility in creating a healthier food supply by stocking, promoting, and reducing costs of healthier foods in their stores. Local food wholesalers were involved in a top-down, participatory approach to develop and implement an effective and sustainable program. This study will provide evidence on the effectiveness of price incentives and health communications, separately and combined, among a low-income urban U.S. population.

**Trial registration:**

ClinicalTrials.gov: NCT02279849 (2/18/2014).

## Background

Obesity is arguably the leading public health problem facing Americans today, contributing to more annual chronic disease-related deaths, disability, and financial burden than either alcohol or tobacco use [1]. Minority groups have a higher prevalence of obesity than whites, and non-Hispanic blacks have the highest prevalence among all ethnic groups in the U.S. [2,3]. Analysis of NHANES data found that low-income groups were also disproportionately affected over a span of 30 years [3-5].

In Baltimore City, Maryland, racial and economic health disparities persist. Within the city, the poorest (< $15,000 annually) groups are 2.4 times more likely to be obese compared to those with the highest incomes (>$ 75,000 annually), while low income neighborhoods have the lowest availability of healthy foods [6,7]. Twice as many black residents live below poverty level (26.7% vs 14.5%), and have almost twice the obesity rate (43.5% vs. 23.3%) as whites, and have the highest rates of death from diabetes, the comorbidity most strongly influenced by body weight, compared to all other ethnic groups in the city [8-10]*.* In the United States, poverty and obesity are positively correlated [11], and though public health programs have limited capacity to affect poverty status, intervening on possible mediators, such as food access, can help to eliminate health disparity and inequity gaps. [12]. In the past decade, improving food environments and increasing access to healthy foods has been identified as a key strategy for obesity prevention and reduction efforts [13].

Low-income, predominantly black neighborhoods of Baltimore City are replete with small convenience-type food stores and nearly void of supermarkets [14]. Small stores are a primary food source among inner city residents [15], which are often lacking healthier foods, including fresh fruits and vegetables, low-fat milk, and whole wheat bread [7]. Sharma et al. [16] reported high consumption of high fat foods and sugar-sweetened beverages, and extremely low consumption of fruits and vegetables, among low-income black residents. Small food store interventions have had positive impacts on store availability, sales, and consumption of healthier foods and beverages [17]. Most small store trials have used education- and communication-based strategies, such as signage and shelf labels, and/or structural changes, such as shelving or refrigeration, to improve food access [17]. However, solely increasing the availability of healthy foods will have limited impact on purchasing and consumption if the foods within these environments are not affordable. To our knowledge, no small food store studies have tested the feasibility or impact of pricing discounts to increase healthy food purchasing and consumption [17].

The price of food is one of the most important determinants of consumer purchasing decisions [18]. A systematic review of field experiments by An [19] demonstrated that direct-to-consumer price discounts were consistently effective in increasing the purchase and consumption of healthier promoted foods. However, most of the studies occurred in larger food venues, such as supermarkets, restaurants, and cafeterias, and only 4 out of 20 studies targeted low-income populations [19]. Furthermore, only three published factorial design trials, designed to show interactions between interventions, have tested health education/communications strategies and pricing reductions, separately and combined, on consumer purchase and consumption of healthy foods in a retail food store-setting [20-22]. A 2x2 randomized controlled trial (‘SHOP’) in 8 New Zealand supermarkets found that nutrition education had no effect on food purchases, while a 12.5% discount in price was associated with 11% increase (p < 0.001) in healthier food purchases in both pricing and combined groups [21]. While these results are promising, this trial did not target low-income consumers, who often have less access to healthier foods and are more sensitive to price changes than their higher income counterparts [23,24]. A 2x2 randomized controlled trial in Dutch supermarkets found no effect of nutrition education alone on fruit and vegetable purchases, but a significant increase in fruit and vegetable purchases with a 50% discount, and the greatest increases when pricing was combined with nutrition education [22]. This trial illustrates the impact of subsidies on purchasing behavior; however, the likelihood of translating such a high subsidy into policy may not be politically feasible, whereas small price changes with adjunctive strategies may be possible [25].

There have been no factorial design pricing and communications trials in small food stores, and none in any type of food store domestically. The strategies, results, and implications of prior food store trials in Australia, New Zealand, and the Netherlands are unlikely to be generalizable to those implemented and observed in the United States. Moreover, all three trials occurred in supermarkets, which have greater economies of scale compared to the small retail stores ubiquitous in poor, urban neighborhoods. Small retail food stores are a predominant food source in Baltimore City and small food store shoppers purchase more unhealthy foods compared to those that use other food sources [15]. However, small independent food stores operate within a different context than do larger food store chains (e.g., limited purchasing power, less infrastructure, independently owned), and we do not know what combination or level of price reductions and communications will spur healthier food purchases and consumption among a lower income and more price sensitive population.

The three published factorial design pricing trials applied direct-to-consumer discounts through vouchers in the mail [22], or electronically at checkout [20,21]. Pricing interventions that subsidize healthier foods for consumers may be effective but also may be costly, and therefore harder to sustain in the long term. For example, evaluation of the Healthy Incentives Pilot, a government funded program that provided financial incentives to SNAP participants for the purchase of healthier food, found significant increases in fruit and vegetable consumption, but also estimated that implementing the program nationwide for five years would cost $90 million, not including incentive costs for retailers [26]. An alternative way to reduce consumer costs of healthier foods is through performance allowances (also known as trade promotions or promotion allowances), a standard food industry marketing practice. With performance allowances, manufacturers pay downstream distributors and/or retailers for a certain performance, such as slotting allowances to acquire prime shelf space or advertising allowances paid from the marketer to the retailer for advertising a certain product. Trade promotions, including performance allowances, are used to increase sales and stocking of certain foods during specific periods of time [27]. In light of increasing public pressure to offset the negative health consequences of their products, the food industry’s self-regulatory efforts could include performance allowances to increase sales and consumption of healthier and lower calorie foods. For example, a manufacturer could provide slotting or advertising allowances on their lower calorie or healthier snacks, which theoretically would help to increase both their supply and demand. This method not only has the potential to create long-term availability of healthier foods at retailers, but supports the notion that food companies should be required to reduce the public health problems (i.e., obesity) for which some public health experts hold them responsible [28]. To our knowledge, no public health intervention trial has employed performance or trade allowances as a pricing strategy to increase healthy food purchases and consumption.

This manuscript describes the study design of a multilevel communications and pricing intervention called B’More Healthy: Retail Rewards (BHRR). In this study, we test the impact of performance-based allowances on the purchase, stock, display, and sales of healthier foods in wholesale and small retail food stores in low-income areas of Baltimore City. We focus on small food stores and a low-income population, where sensitivity to price changes are greater and where food access research is needed most [23,24]. Lastly, we introduce an innovative approach that incorporates established and effective food industry practices within a public health framework. Descriptions will follow the Consolidated Standards of Reporting Trials (CONSORT) reporting guidelines.

### Study aims

The overarching goal of the BHRR trial is to develop, implement, and evaluate a multi-level communications and pricing intervention to improve the food environment in low-income areas of Baltimore City, Maryland. BHRR has three primary aims: (1) to conduct formative research with representatives of multiple levels of the Baltimore food environment (i.e., local wholesalers, retail food store owners, and consumers) in order to select key foods for promotion, and determine appropriate communication strategies and price reductions, (2) to implement a multi-level program with two local wholesale stores, and twenty-four small food stores and their customers, and assess program implementation through detailed process evaluation, and (3) to assess the impact of separate and combined pricing and communication strategies on consumer food behaviors, mediating psychosocial variables (i.e. self-efficacy) and weight outcomes; small store healthy food stocking, healthy food sales, and mediating storeowner psychosocial factors; and wholesaler sales and stocking of selected healthy foods.

## Methods

### Study design

**Figure 1:**
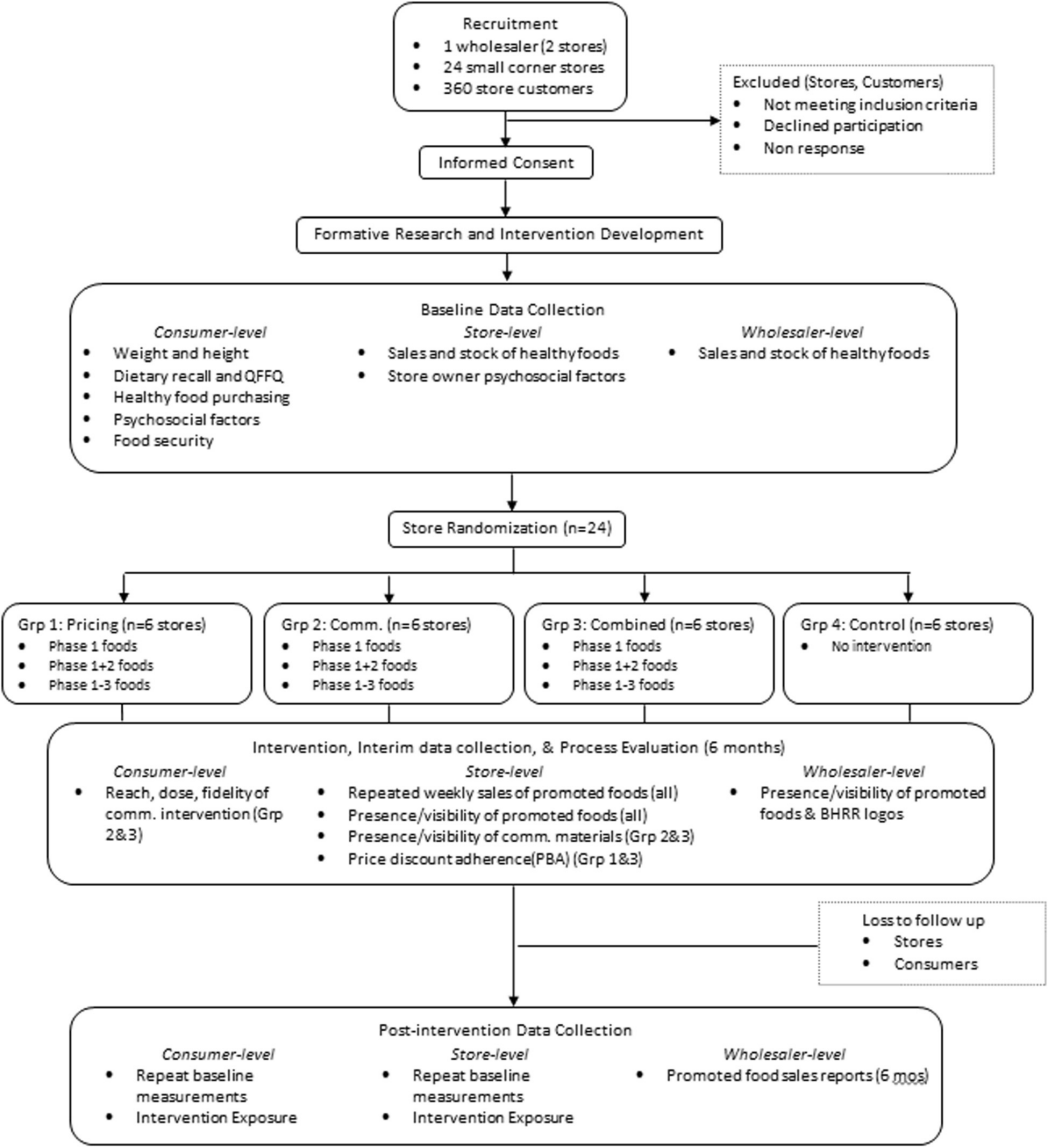
**BHRR study design.**

BHRR is a 2x2 factorial RCT (Figure 1). Twenty-four small corner stores located in low-income census tracts of Baltimore City were randomized to one of four treatment groups: communications only (n = 6), pricing only (n = 6), combined communications and pricing (n = 6), or control (n = 6). Performance allowances in the form of healthy food discounts (10-30% off wholesale price) were directed from the wholesaler to the pricing only and combined intervention stores (12 stores total) at checkout for 6 months. In return for the discounts, storeowners were asked to stock selected healthier foods, and display communications materials and/or pass discounts to their consumers. The communications only and combined intervention stores (12 stores total) received in-store health communications, including taste tests, posters, and small refrigerators or freezers, to help stock and promote the sale of selected healthier foods. All customers of the participating corner stores were exposed to the 6-month intervention directed to that store. This study was approved by the Johns Hopkins Bloomberg School of Public Health Institutional Review Board.

### Study hypotheses

The study tests the consumer-level hypotheses that, by the end of the 6-month intervention, customers of the 18 intervention stores (pricing only, communications only, and combined) will have, 1) greater increases in frequency of purchasing and consumption of the promoted foods than those at control stores, with the greatest increases among those consumers at combined intervention stores, and 2) greater increases in psychosocial factor scores relating to healthy food choices than those at control stores, with the greatest increases in the combined intervention stores. The study tests the store-level hypotheses that, by the end of the 6-month intervention, the 18 intervention storeowners will have 3) greater increases in sales and stocking of promoted foods compared to control stores, with the greatest increases in the combined intervention stores, and 4) greater increases in store owner psychosocial factor scores related to stocking and sales of promoted foods compared to control stores, with the greatest increases in the combined intervention stores.

### Theoretical framework

The theoretical framework that guides the BHRR intervention and its evaluation is based on Social Cognitive Theory (SCT) [29], the Social Ecological Model (SEM) [30], and economics’ law of demand. SCT and SEM stress that individual behavior change relies on the dynamic interplay between the individual and his or her environment, and in order to create sustained change, public health interventions must target multiple levels. They have been extensively employed for diet-related and store-based interventions [17,31]. Small refrigerators or freezers were supplied in order to create supportive environments to stock and promote healthier foods in small stores. Individual behavior change using health communications was sought through intervening on possible mediators to stocking and purchasing healthier foods such as self-efficacy, intentions, and outcome expectations. Baseline analyses on the association between psychosocial factors and food acquisition behaviors have found that higher self-efficacy and intentions scores are associated with greater frequency of healthy food purchases and lower frequency of unhealthy food purchases (unpublished data). Thus, targeting specific psychosocial factors in health interventions may enhance an individual’s ability to make healthier food choices.

In economics, the law of demand states that, all else being equal, there is an inverse relationship between quantity demanded and its price. We expect that a reduction in price of healthier foods will elicit an increase in consumer purchase and consumption of promoted foods. Monetary performance allowances were provided as incentives for wholesalers and retailers to stock and discount healthier foods and thereby increase supply and demand.

### Setting

Baltimore City’s overall population is approximately 622,000; where almost one quarter live below poverty level, and almost two-thirds are African American [32]. There are approximately 659 small retail food stores^a^ within city limits, many of which are located in food deserts^b^ [14]. A 2007 community food assessment found that 46% of monthly shopping trips among residents of Southwest Baltimore were to small food stores, where average individual expenditures were $114 per month [33]. The target group in BHRR is low-income African American adult customers of small retail stores located in the city.

Food wholesalers sell larger quantity goods to industrial, institutional, and commercial users, but generally do not sell in large amounts to individual consumers. Two competing businesses operate three wholesale stores or warehouses located within Baltimore City limits, where small food retailers can pick up items. Two stores are located in the southwest region of the city, and one store is located in the northeast section of the city. BHRR works with one wholesaler, which operates two warehouses that serve retail stores in the Baltimore market. One of the warehouses also serves as a distribution site for a direct delivery service. The warehouses carry over 30,000 items, including National Brands (Deer Park, Pepsi, Frito-Lay), private labels (Richfood, Everyday Essentials), and regional items (Esskay, Rutters, Utz, Everfresh). Small storeowners represent 90% of its clientele, while the other 10% are foodservice customers.

### Eligibility and recruitment

#### Wholesaler recruitment

Both wholesale businesses in Baltimore City were invited to participate in the study. One wholesale business (with a single store location) declined participation. All of the 24 participating retailers regularly shop (1x/week) in at least one of the participating wholesaler’s store locations. In addition, 16 out of the 24 participating storeowners use the other wholesale business regularly, 19 use a warehouse club located outside of city limits (and not considered for the study), and 13 use a discount department store. The participating wholesaler has agreed to provide research staff with sales data pertinent to the study.

#### Store recruitment

The Johns Hopkins’ Center for a Livable Future provided study staff with GIS maps of small food stores that are located in low-income census tracts where greater than 75% of residents are African American. Study staff selected stores on the maps that met the following inclusion criteria: 1) in 2009, had average annual purchases of $5,000-20,000 from one or more participating food wholesalers; 2) not part of past store-based intervention trials in Baltimore [34,35]; and 3) were at least ¼ mile apart from each other. Recruitment of storeowners involved explanation of the purpose of the study, and distribution of recruitment materials explaining frequently asked questions and answers about the program. Korean-speaking research staff and translated recruitment materials were used in the recruitment of Korean storeowners. Staff approached 82 active stores for participation in the study; 34 storeowners refused to participate (e.g., citing a lack of time or not providing a reason), 16 asked staff to return when the owner was there, and 32 initially agreed to participate and out of those, 8 dropped out of the study prior to baseline data collection. Twenty-four storeowners completed surveys at baseline, 23 storeowners completed post-intervention surveys, and 22 storeowners completed the 6-month intervention in its entirety. Written informed consent was obtained immediately preceding any interviews and surveys.

#### Consumer recruitment

A convenience sample of the first fifteen eligible customers/consumers that expressed interest in participating in the research study were recruited between May and September 2012 (total n = 360 consumers). Participants were eligible for the study if they: (1) were African American adults aged 21 or older, (2) lived within 0.25 miles of the store where they were recruited, (3) shopped in the store at least once a week, and (4) were the main food shopper for their household. All participants were interviewed outside of the stores where they were recruited, were explained the purpose of the study and signed a written informed consent form prior to interviews.

Both store owner and consumer respondents were compensated with $20 gift cards upon completion of each interview, which lasted approximately 1 hour.

### Power calculation

Data used to calculate sample size were taken from a previous store-intervention study of African American adults in low-income inner city areas of Baltimore [35]. To account for clustering, we calculated sample size based on the intra-class correlation (ICC) formula [36] using a previous study’s psychosocial and food purchasing data. With a final, post-intervention sample of 12 consumers per store (n = 288 total) and 6 stores per groups, we will be able to detect an increase of 2 points in the food knowledge score, which implies that the respondent can correctly answer 2 additional questions related to food knowledge; an increase of 5 points for the self-efficacy score, which implies that the respondents feels confident to perform at least 1.3 additional healthful behaviors (i.e., choosing water instead of a sugar-sweetened beverage); and an increase in 3 points on healthy eating intentions, which implies that the respondent intends to perform at least 1 additional healthful behavior (i.e., purchasing 1% milk instead of whole milk). Using a conservative estimate of ICC for healthy and unhealthy food getting frequencies, we will be able to detect approximately 20 points increase in the healthy food getting frequency and a 20 point decrease in the unhealthy food getting frequency.

### Randomization and blinding

To ensure comparison of treatment groups with similar characteristics, stores were stratified by WIC status and daily sales volume. Daily sales volume was calculated from the baseline unit sales of promoted foods in the past 30 days. Greater than or equal to 20 unit sales of promoted items per day was defined as large volume, while less than 20 unit sales per day was defined as low volume. Sales volume was used as a proxy for daily sales revenue, since storeowners were reluctant to share exact monetary estimates with research staff. Similarly, WIC status was used as a proxy for healthy food stocking, since stores carrying WIC must have a minimum required stock of certain healthy foods at all times. Thus, randomization was stratified by: high-volume stores with WIC; high-volume stores without WIC; low-volume stores with WIC; low-volume stores without WIC.

### Intervention design and implementation

Extensive formative research, including in-depth interviews, observations, and focus groups with small storeowners and consumers, was carried out from January to October 2012 and is summarized in Table 1. Qualitative data was transcribed, entered and coded using the Atlas-Ti textual data analysis software program (version 7.0, Scientific Software Development GmbH, Berlin, 2012). In addition, multiple structured business meetings with wholesale staff helped to formulate appropriate pricing strategies and protocol for passing on discounts to customers.

**Table 1:** **Completed formative research with wholesalers, storeowners, and consumers**

**Date**	**Level**	**Activity**	**Objectives**
**Jan – Mar 2012**	Wholesaler	Direct Observation (n = 12)	To examine retailers’ purchasing patterns and food selections and to understand marketing factors influencing their choices at wholesale stores.
**Feb – Mar 2012**	Store owner	Direct Observation (n = 17)	To create store maps, highlighting how items are stocked and displayed. To observe any existing in-store promotions, including pricing or communications marketing strategies. To observe customers’ shopping patterns and purchases.
**Mar 2012**	Store owner	Participant Observation (n = 1)	To shadow specific retailers that also completed an interview as they shopped at the wholesaler, to examine shopping patterns and to further understand retailers’ perceptions of food choices and availability.
**Mar – Apr 2012**	Store owner	In-depth Interview(n = 17)	To understand stocking decisions, barriers and facilitators to stocking healthier food products, relationships with customers and suppliers (e.g., wholesalers, vendors), pricing determinants, promotional strategies, and business infrastructure (e.g., Korean American business owner networks).
**Mar – Apr 2012**	Consumer	In-depth Interview (n = 9)	To explore healthy food preferences and perceptions, food sources, purchasing decisions at corner stores, and motivators/facilitators to increase healthy food purchasing in corner stores.
**Mar 2012, Oct 2012**	Consumer	Focus Groups (n = 2, 11 and 12 consumers, respectively)	To discuss potential promoted foods, healthy food perceptions, healthy food availability, corner store shopping experiences, relevant words or phrases denoting ‘healthy’ that may appeal to the consumer, strategies to increase healthy food purchasing, and feedback on study logo design. The second focus group served to refine acceptable promoted food items (via taste testing and discussion), key messages/communications formats, and acceptable price ranges to increase healthy food purchasing in corner stores.
**May 2012**	Consumer	Pile sorting and ranking (n = 33)	To identify and refine foods and beverages for promotion. Staff collected proximity and ranking data on 24 potential promoted foods/beverages. Individual items were first free-sorted into groups by each consumer. Consumers were then asked to sort foods/beverages into 3 groups: very interested to eat, somewhat interested to eat, not going to eat.
**Jan 2012 - Feb 2013**	Wholesaler executives	Intervention planning meetings (n = 10)	To implement stocking of new promoted foods, to refine acceptable promoted food items, to develop sustainable pricing strategies based on price sensitivity to increase healthy food sales, and to develop a protocol for applying healthy food discounts to the pricing intervention groups.

#### Selection of foods for promotion

Promoted items included a combination of fruits, vegetables, low-fat snacks, lower calorie beverages, and whole grain products. The items were intended to serve as healthy alternatives for items most frequently purchased from corner stores, including nutrient-poor, calorie-dense snack foods and drinks (i.e., chips, cookies, sodas) and staple food items (i.e., bread, cereal, milk, cheese). Promoted foods fell into one or more categories as defined by the U.S. Food and Drug Administration (FDA) [37]:

- Low-fat – 3 g or less per Reference Amount Customarily Consumed (RACC) or 100 g and not more than 30% of calories from fat
- Reduced fat – at least 25% less fat per RACC or 100 g
- Reduced sugar – at least 25% less sugar per RACC or 100 g
- Low-calorie – 40 calories or less per RACC or 120 kcal or less per 100 g
- Reduced calorie – at least 25% fewer calories per RACC or 100 g
- High-fiber – contains 20% or more of the Daily Value for fiber per RACC.

The intervention consisted of three phases, each of which expanded upon the preceding phase, so that by the final phase, all foods and beverages were promoted simultaneously. Phase 1, from February to April 2013, promoted lower calorie/fat beverages including 1% milk, bottled water, and selected reduced calorie colas. Phase 2, from April to June 2013, promoted nutrient-dense staple foods including 100% whole wheat bread, canned tuna in water, and frozen vegetables, in addition to Phase 1 drinks. Phase 3, from June to August 2013, promoted lower fat snack foods; including fresh fruit, low fat granola bars, and baked potato chips, in addition to Phase 1 & 2 foods.

#### Pricing intervention

A previous Baltimore-based corner-store study described the importance of addressing both financial risk of stocking new products and the psychosocial burden that many storeowners feel in response to the pressure of stocking promoted foods [38]. BHRR was designed to reduce this burden by allowing storeowners to purchase healthier promoted items at reduced costs from the participating wholesaler. The amount of discount applied to each promoted item was determined through formative research with wholesale staff (as the minimum discount required to result in increased sales), and with storeowners (as the minimum discount required by retailers to agree to stock the foods and pass through savings to customers (known as channel or retail pass-through). Discounts ranged from 10-30% and were similar to amounts applied in previous studies [20,22,39]. Discounts on promoted items were automatically applied at wholesale registers to stores receiving the pricing intervention (n = 12) from February to August 2013. Grant funding was used to reimburse the wholesalers. In exchange for discounts, the pricing group storeowners (Groups 1 & 3) agreed to the terms of the performance allowance: to stock the promoted foods, to provide retail pass-through to customers, and to display communications materials (combined group only). Storeowner compliance to the performance allowance strategy was monitored throughout the program through process evaluation. Item discounts were introduced at each phase and sustained for the duration of the program so that during the first month only beverages were discounted, and by the last month, all promoted foods were discounted simultaneously.

#### Communications intervention

##### Wholesaler-level

The communications portion at the wholesaler-level was minimal due to the necessity to prevent cross-contamination of store owners in the pricing and control groups (Groups 1 & 4). ‘Hidden’ communications for Groups 2 & 3 (Communications & Combined groups) included 1) marking promoted foods and beverages with a 2′ circumference BHRR logo sticker at both Cash & Carry locations, and 2) providing intervention store owners with a pamphlet that contained exact aisle locations for the items. Wholesale staff members were instructed to keep the promoted items stocked at all times during the intervention period.

##### Store-level

A graphic artist and research staff developed store-level communications materials based on formative research findings. Preferred words and phrases cited by corner store customers included ‘energy’, ‘living better’, ‘clean and fresh’, ‘natural’, ‘fresh foods at a reasonable price’, and ‘100%’ (as in whole grains). Other suggestions for point of purchase materials were to provide quick descriptive words that explained why a particular food was healthy (i.e., fiber-rich, heart-healthy), as well as quick, catchy sayings (i.e., ‘refresh!’, ‘power up!’) to appeal to consumers. Posters and window signs were requested to be ‘simple’ and ‘easy to read’ since the amount of time customers spent in the store was brief. The colors purple and orange were used in all communications materials and were selected to match Baltimore City professional football and baseball teams. Materials were piloted in the community and revised before intervention implementation to ensure acceptability and resonance.

For the communications stores (Groups 2 & 3), each phase included 4–5 visits to stores for interactive sessions that included giveaways, educational handouts or recipe cards, and taste tests or educational activities. Promotional materials were tailored to each phase’s theme. For example, in-store promotions for Phase 1: Beverages, included blind taste testing of lower calorie beverages, an educational display showing the amount of sugar in commonly consumed drinks, and free drink tumblers with the project logo. Posters displayed the benefits of switching to water or a low-calorie drink, and shelf labels and talkers highlighted promoted items on the shelves. The communications stores also received a small refrigerator or freezer to store fresh or frozen fruits and vegetables and other healthy foods as an additional incentive.

#### Interventionist training

A BHRR manual of procedures was developed and used to train interventionists and to standardize practice across field sites. A 2-day interventionists’ training, led by the study coordinator, included nutrition education sessions, demonstrations and role-play, prior to intervention implementation. Weekly staff meetings served to address issues associated with program implementation.

#### Data collector training

Before beginning the study, data collectors completed a computer-based course in the protection of human research subjects (CITI Program, University of Miami). Each data collector also participated in a 2-day, in-person, data collector training program led by the Principal Investigator, which reviewed: 1) human subjects ethics principles and procedures, 2) recruitment, sampling and consenting procedures, and 3) instruments and protocol for delivery. Data collectors were trained using a combination of lectures, role-play, and supervised practice interviews.

### Outcomes and measures

Outcomes were assessed at a minimum of two time points. Baseline interviews were conducted with small storeowners and consumers from April to December 2012. Post-intervention interviews were conducted from November 2013 to March 2014. All interviews were conducted in a quiet setting in or near corner stores, in participants’ homes, or at the Johns Hopkins Bloomberg School of Public Health. Interviews with storeowners whose primary language was Korean were conducted by Korean-speaking research staff. English versions of forms were used for all data collection. A summary of study measures is shown in Table 2.

**Table 2:** **Summary of study measures**

**Measures**	**Instrument**	**Baseline**	**Interim**	**Post-intervention**
**Impact**				
*Consumer-level*				
Food acquisition^1^	AIQ	✓		✓
Food-related psychosocial factors^1^	AIQ	✓		✓
Food source use	AIQ, 24-hour dietary recall	✓		✓
Health beliefs & attitudes	AIQ	✓		✓
Food Assistance participation	AIQ	✓		✓
Socio-demographics	AIQ	✓		✓
Household food security	AIQ	✓		✓
Weight	AIQ	✓		✓
Height	AIQ	✓		✓
Promoted food consumption^2^	QFFQ	✓		✓
Diet	24-hour dietary recall	✓		✓
*Store-level*				
Stock of promoted foods^2^	SIQ, Environmental Assessment	✓	✓	✓
Sales of promoted foods^2^	SIQ, Weekly sales recall	✓	✓	✓
Food-related psychosocial factors^2^	SIQ	✓		✓
Store operations	SIQ	✓		✓
Customer & employee attributes	SIQ	✓		✓
Food acquisition & promotion	SIQ	✓		✓
*Wholesale-level*				
Sales of promoted foods	Wholesale sales records	✓	✓	✓
**Process evaluation**				
Dose delivered, reach, fidelity of consumer communications (e.g., interactive sessions)	Interventionist PE form		✓	
Dose received of consumer communications and pricing components	Consumer intervention exposure form			✓
In-store communications strategies (store, wholesaler)	Environmental assessment, Wholesale PE form	✓	✓	✓
Healthy food availability & visibility (store, wholesaler)	Environmental assessment, Wholesaler PE form	✓	✓	✓
Price discount implementation (store, wholesaler)	Environmental assessment, Wholesale sales records	✓	✓	✓
Dose received of store owner communications and pricing components	Store intervention exposure form			✓
Small store environment & infrastructure	Environmental assessment	✓	✓	✓

^1^Primary outcome.

^2^Secondary outcome.

#### Primary outcomes

The primary outcomes of interest are the average change in consumer purchase of promoted foods and beverages, and related consumer psychosocial variables across treatment groups from baseline and post-intervention. The Adult Impact Questionnaire (AIQ) was used in past Baltimore Healthy Stores trials and was modified for this study [34,40,41]. Included in the 174-question AIQ is a section that assessed the frequency of food purchasing or ‘food getting’ (food obtained without purchasing) for 37 foods or food groups in the past 30 days, including promoted foods and unhealthier counterparts (e.g., baked chips vs. regular chips). The AIQ also contains sections that addressed individuals’ psychosocial factors, including self-efficacy, intentions, and knowledge to perform healthy eating behaviors. The self-efficacy section contained 10 questions that captured the respondents’ self-confidence in making healthy food choices. For example, respondents choose out of four responses ranging from “very easy” to “would be impossible” to questions such as, “How easy or difficult would it be for you to eat fresh or frozen vegetables every day?” The 10-question intentions section addressed respondents’ intentions to purchase, consume, and prepare foods promoted by the intervention using a forced-choice format (i.e., “The next time you buy a sweet snack, which will you choose, Donut, Granola Bar, or Tastykake?”), and the 10-question knowledge section tested the ability to answer nutrition-related questions, such as interpreting food labels.

#### Secondary outcomes

##### Consumer dietary intake and consumption of promoted foods

Promoted food consumption was assessed using a previously-fielded brief quantitative food frequency questionnaire (QFFQ). Participants were asked to report the frequency of consumption of 22 foods/food groups over a 30-day period, choosing from eight categories ranging from “never” to “two or more times per day”. In addition to the QFFQ, a single quantitative 24 hour dietary recall was collected using a 5-step multiple pass methodology [41]. The dietary recall and QFFQ were collected on both weekdays and weekend days. The instrument was modified to include consumer food sources (i.e., supermarket, farmer’s market, corner store). Evaluation of consumer exposure to specific food sources will allow staff to track impact of local food policy initiatives (i.e., the proportion of calories consumed from urban corner stores). Dietary data will be analyzed using Nutrition Data System for Research (NDSR) software (version 11: Nutrition Coordinating Center, University of Minnesota).

##### Storeowner psychosocial variables to stock/sell promoted foods

Changes in psychosocial constructs toward the stocking and sales of promoted food items were assessed with the owners of participating corner stores. The Store Impact Questionnaire (SIQ) was adapted from an instrument previously used in former small store interventions [35,42] and was piloted before baseline data collection. The SIQ included sections on outcome expectations on sales of healthy foods and beverages, self-efficacy to stock, promote, and sell healthy foods and beverages, and intentions to sustain stocking and promotions on healthy items. Respondents were read a series of statements and asked to choose from one of five answers: Strongly Agree, Agree, Undecided, Disagree, or Strongly Disagree. Outcome expectations for promoted food sales was assessed with 16 questions (i.e., “Baked potato chips will sell well in my store”); outcome expectations on overall program impact was assessed with 18 questions (i.e., “If I receive a produce refrigerator for my store, fresh fruit/vegetable sales will increase”); 15 questions each were included to evaluate self-efficacy for stocking promoted foods (i.e., “I can stock 100% whole wheat bread in my store”) and intentions to sustain stocking of promoted foods (i.e., “I will stock frozen vegetables in my store after the program is completed”); and 6 questions assessed storeowners’ intentions to sustain pricing or communications promotions on the promoted foods after the program’s completion (i.e., “I plan to display BHRR promotional materials even after the program is completed”).

##### Sales of promoted foods

The SIQ captured promoted food sales by asking each participating store owner to estimate the number of units (i.e., cans, packages) of 15 key promoted foods sold in the store per day over the last 30 days. Additionally, a sales recall instrument, which has been used in earlier Baltimore-based studies, recorded store sales bi-monthly during the trial by asking each storeowner how many units of each promoted item were sold in the past 7 days [35]. A total of 12–15 weekly sales recalls were collected per store.

#### Other outcomes

##### Consumer body mass index

Anthropometric measurements were taken with adult consumer respondents wearing light, indoor clothing at baseline and post-intervention. Body weight was measured to the nearest 0.1 pound with Seca Model 880 portable electronic scale (Seca Corporation, Columbia, MD). Standing height to the nearest 1/8 (0.125) inch was measured with a Shorr Height Board (Shorr Productions, Olney, MD). Weight and height measurements were taken twice and averaged. If height measurements differed by ≥ 0.25 in or weight differed by ≥ 0.2 lb., a third measurement was taken and all 3 were averaged. These measures were used to calculate adult body mass index (BMI).

##### Household food security, food assistance, health beliefs, socio-demographics

The AIQ included the 18-item Household Food Security Survey (HFSS) module (Economic Research Service, USDA, 2008). The 18-item section included 10 questions that concern the experiences of adults and 8 concerning respondents’ experiences of providing food to children in their households. A section with 13 questions assessed participants’ health beliefs and attitudes and body image (e.g., “Healthy foods are expensive” and “I am satisfied with my weight”) using a 5-point Likert scale. Also included were questions regarding food assistance program participation over the past 12 months (e.g. SNAP, WIC), education level, income, employment, marital status, and housing.

##### Wholesaler sales of promoted foods

The participating wholesaler has agreed to provide promoted food sales records generated from wholesale databases. Reports will provide information on unit sales and revenue for each promoted food or beverage item overall and per participating store for each promoted food item from January to September 2013.

#### Process evaluation

Intervention implementation at the consumer-level was monitored twice monthly. Data collectors evaluated interactive sessions at each of the communications stores (n = 12) by reporting the number of consumers contacted through interactive sessions (reach), the number of different intervention components (i.e., giveaway, taste test, pamphlet, recipe card) delivered to each consumer at each interactive session (dose delivered), and how well each interactive session was delivered (fidelity) [34,43].

Intervention implementation at the store-level was monitored using a store environmental assessment form, modeled after the Nutrition Environment Measurement Survey-Stores (NEMS-S) instrument [44], to assess presence and placement of promoted food items and communications materials, and to provide additional commentary on contextual factors (e.g. cleanliness of store, expired items). The form also assessed whether the price was marked and if BHRR shelf labels or talkers were present and correctly identified the item. A wholesaler process evaluation form was used to track presence and visibility of promoted food items and BHRR logos. Pricing discount implementation was monitored using wholesaler electronic sales records and a weekly sales recall for small stores (12–15 sales recalls per store). At post-intervention, a separate intervention exposure questionnaire measured dose received, defined as the proportion of respondents who successfully recall exposure to a variety of specific intervention components/materials, for storeowners and customers.

### Analyses

Descriptive statistics will be used to compare the demographic characteristics of intervention and control participants at baseline using means or medians for continuous variables and proportions for categorical variables. A series of scales and scores will be developed to evaluate the impact of the intervention on food acquisition and psychosocial factors for consumers and storeowners [35,40]. All scales will be assessed for internal consistency and reliability using Cronbach’s alpha.

Key outcome variables will be store- and consumer- level outcomes, with treatment condition as the primary exposure variable. An intent-to-treat approach will be used to test study hypotheses. Multiple regressions will be conducted to assess program impact on consumer food-getting and food consumption, consumer BMI and food security, consumer and storeowner psychosocial variables, and small store purchasing and sales. All analyses will account for clustering (by store for individual-level outcomes and over time for store-level outcomes) using multiple regressions with clustered robust standard errors (e.g. Huber-White), generalized estimating equations (GEE), or multilevel modeling methods [45]. We will first test for interactions between pricing and communications intervention groups, and will remove the interaction term if effects are not found [46]. Statistical tests will be two-tailed with an alpha set at 0.05. Summary statistics will be used as appropriate for process evaluation data.

## Discussion

Utilization of food industry sales promotion techniques to improve healthy food purchase and consumption is a novel approach. To date, pricing research has centered on consumer promotions, either through deals offered by manufacturers directly to consumers, or by retailers to consumers [27]. Policy-driven pricing initiatives to improve food behaviors have been in the form of consumer subsidies or taxes (e.g., WIC vouchers). In contrast, performance allowances are deals offered (i.e., discounts, rebates, coupons) to retailers with the expectation that retailers pass them through trade deals to consumers [27]. Retailers may benefit from performance allowances on healthy foods by either, buying at discounted prices and selling at normal prices, or by increasing sales of the promoted item when savings are passed on [27]. Both strategies increase the availability and potential sales of healthier foods in stores, whereas consumer price incentives may not motivate retailers to stock those foods that are healthier for consumers. Consumer food preferences and norms are heavily influenced by food industry advertising and sales promotions. With sufficient incentives (i.e., tax breaks, regulatory action), food manufacturers can help to increase the demand for healthier products and behaviors that may help towards the reduction of obesity and its related co-morbidities.

Another distinguishing innovative characteristic of the study is that this is the first randomized controlled trial to involve food wholesalers in a food access intervention program. One previous study conducted phone interviews with produce wholesalers in New Orleans [47], however, none to date have implemented a research study with food wholesalers. In addition, while a few other cities have partnered with distributors and wholesalers in addressing healthy food access [48], this is the first program to do so in Baltimore. Given that wholesale stores are the main sources of food for small retail stores in the city, it is both intuitive and essential to involve these suppliers in healthy food access initiatives. An obvious approach to increasing healthy food supply in small corner stores is to ensure adequate stock of healthy foods at their wholesalers. However, simply stocking healthy foods does not guarantee that the foods will be bought, thus, more complex pricing and promotional strategies to increase demand are being tested in this trial.

The feasibility of using performance allowances and communications will be evaluated through process evaluation. A top-down price promotion may not reach the consumer level, and it is unknown whether simply stocking and promoting the product is sufficient to impact consumer food behaviors or if additional consumer-level price reductions are needed to generate increased demand. Thus, in addition to the study’s main outcome measures, this study will also shed light onto the mechanisms of trade promotions and analyze overall system-level effects (consumer-retailer-wholesaler) (e.g., Do price reductions need to reach the consumer to increase demand for healthier food in low-income urban settings?). Most research on trade promotions, including performance allowances, remains theoretical and overly simplified, using simulations and modeling to determine effects [27]. Therefore, a key research question is whether retail pass-through is a feasible and effective approach to increase healthier food purchases and consumption in a small store setting.

Results will provide original evidence on the effectiveness of multi-level pricing and communications interventions to improve food access in low-income minority settings, and will provide insight for further studies seeking to work with food suppliers and trade promotions to improve the food environment. Such food access interventions, aimed at ultimately reducing the prevalence of obesity among low-income urban populations, may greatly decrease rates of chronic disease and health care costs nationally [49].

## Endnotes

^a^Small food stores are defined as follows: “Superettes,” sometimes called “mom & pop” stores or corner stores, carry a basic, narrow selection of food items. They tend to have few if any service departments, and have annual food sales of less than $2 million. “Corner Stores” are non-chain Superettes in Baltimore City that sell a limited selection of non-perishable food items. Typically operated by the owner or the owners’ family members or friends, “Behind Glass Corner Stores” are characterized by having barriers of Plexiglas walls separating the consumer on one side from the retail items and owner/workers on the other side [14].

^b^Food desert is defined as an area where the distance to a supermarket is more than one quarter of a mile; the median household income is at or below 185% of the Federal Poverty Level; over 40% of households have no vehicle available; and the average Healthy Food Availability Index score for supermarkets, convenience and corner stores is low (measured using the Nutrition Environment Measurement Survey) [14].

## Abbreviations

BHRR: B’More Healthy: Retail Rewards
AIQ: Adult impact questionnaire
PE: Process evaluation
SIQ: Store impact questionnaire
QFFQ: Quantitative food frequency questionnaire
WIC: Special supplemental nutrition program for women, infants, and children
SNAP: Supplemental Nutrition Assistance Program
NHANES: National Health and Nutrition Examination Survey
